# Supplementary material for: Phase-Dependent Differential In Vitro and Ex Vivo Susceptibility of Aspergillus flavus and Fusarium keratoplasticum to Azole Antifungals
Source: J Fungi (Basel). 2023 Sep 26;9(10):966. doi: 10.3390/jof9100966 (PMC10608098; doi:10.3390/jof9100966)
Supplement: Supplementary file 1 [file jof-09-00966-s001.zip › jof-2603580-Supplementary File S2.pdf]

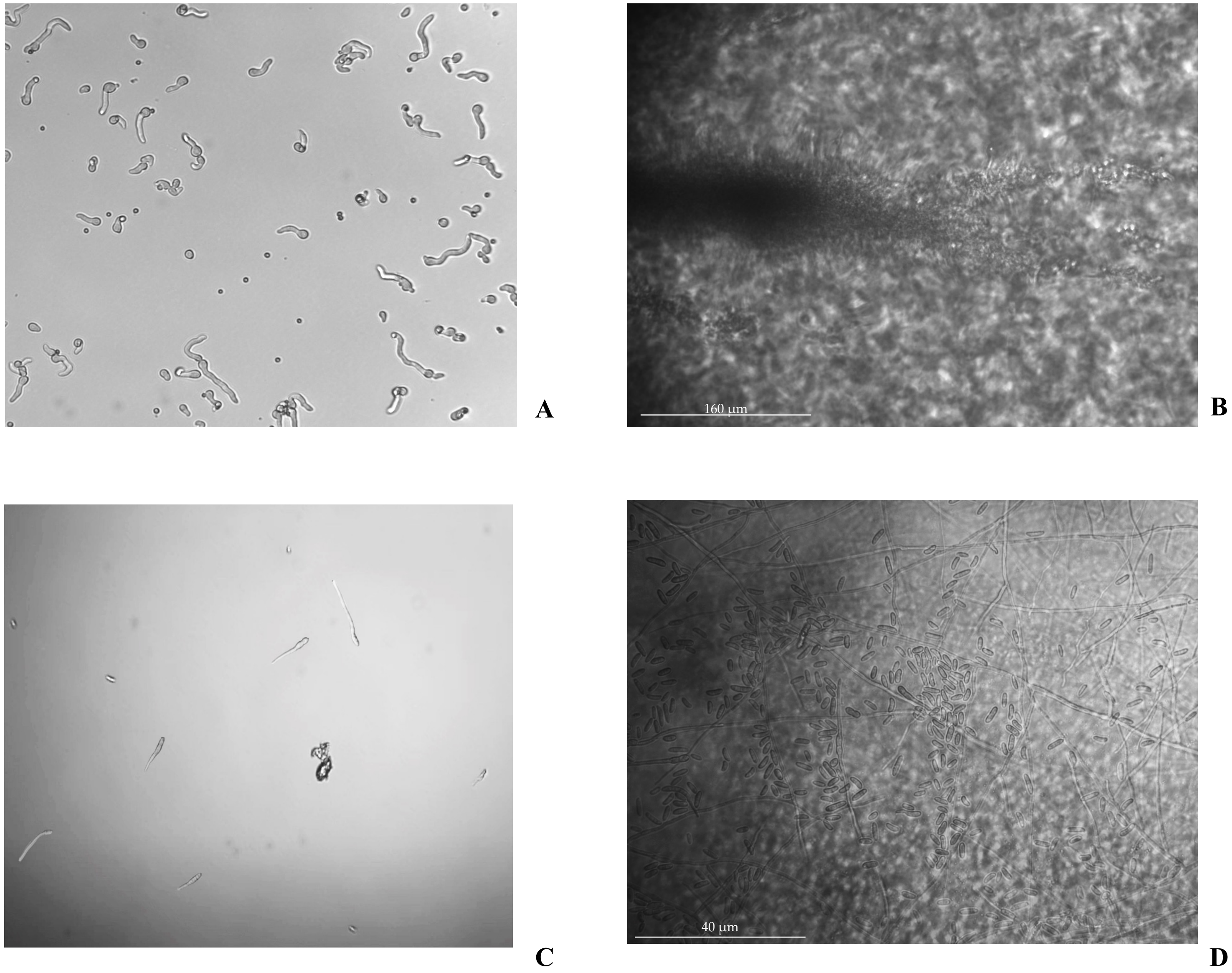

S2. Representative images of germinating conidia of *Aspergillus flavus* conidia on agar (A) and intrastromal (B), and *Fusarium keratoplasticum* conidia on agar (C) and intrastromal (D).
